# Clinical profile of 102 patients with oral lichen planus in Thailand

**DOI:** 10.4317/jced.55814

**Published:** 2019-07-01

**Authors:** Ruchadaporn Kaomongkolgit, Pissacha Daroonpan, Weeraya Tantanapornkul, Jadesada Palasuk

**Affiliations:** 1 Department of Oral Diagnosis, Faculty of Dentistry, Naresuan University, Phitsanulok, Thailand; 2 Department of Restorative Dentistry, Faculty of Dentistry, Naresuan University, Phitsanulok, Thailand

## Abstract

**Background:**

Oral lichen planus (OLP) is a chronic inflammatory disease of the skin and mucous membrane presented with various clinical appearances. The aim of the present study was to elucidate the clinical profile of patients with OLP.

**Material and Methods:**

The dental records of 102 patients who visited Oral Medicine Clinic, Dental Hospital, Naresuan University during 2002-2018 were retrospectively reviewed.

**Results:**

There were 75 (73.5%) women and 27 (26.5%) men, giving a female to male ratio of 2.8:1. The age of OLP patients ranged 20-81 years old with the mean age of 56.4 ± 13.2 years old. Seventy-eight patients (76.5%) had the history of systemic diseases and hypertension was the predominantly one. Most patients were non-smokers (98%), non-drinkers (86.3%) and non-betel nut chewers (98%). The atrophic form (93.1%) was the most common OLP. The lesions were mainly symptomatic (92.2%) and involved multiple locations (67.6%) where the buccal mucosa (79.4%) primarily affected. Only 2% were extraoral lesions detected on the skin. Patients had no family history of OLP or malignant transformation. Ninety-one patients (89.2%) were treated with topical steroid and only 4 patients were prescribed a combination of tropical and systemic steroid.

**Conclusions:**

The results of the study indicated that most of characteristics are in accordance with previous studies. Since, OLP is a chronic inflammatory oral mucosal disease with high recurrence rate, early detection, accurately diagnosis, and long-term follow-up are necessary to evaluate the exacerbation and malignant transformation.

** Key words:**Clinical profile, demographic, oral lichen planus, retrospective study.

## Introduction

Oral lichen planus (OLP) is a chronic inflammatory mucocutaneous disorder affected 0.1% to 4% of general population (1). Although the etiology and pathogenesis of OLP is not completely understood, OLP is believed to be significantly associated with immunological system (2). It is more common in women than men with different age range (3,4). Clinically, OLP can be observed with different clinical appearance. Originally, it was classified into 6 forms as reticular, papular, plaque-like, atrophic, erosive and bullous (4-6). Later, this classification was simplified and divided into reticular, papular, atrophic and erosive lesions, or classified as simply as white and red forms (1). Since the classification is mainly based on clinical appearance, multiple forms can be seen in one patient. The primary affected sites of oral OLP is buccal mucosa, tongue and gingiva which can be presented as symmetrical, bilateral or multiple lesions (1,5). The oral lesions are often asymptomatic but the atrophic and erosive forms can cause symptoms ranging from burning sensation to severe pain. These lead to difficulty in speaking, eating and swallowing (6,7). The OLP patients may develop extraoral lesions on skin, nails or other mucosal surfaces (1). More importantly, OLP may be occasionally concomitant with the extraoral site involvement and oral cancer risk (6). One of the most serious complication of OLP is the development of oral squamous cell carcinoma (OSCC). As a consequence, World Health Organizations (WHO) classified OLP as oral potentially malignant disorders (OPMDs) (8). Although several studies concerning the clinical profile of patients with OLP have been undertaken elsewhere, those information of OLP patients in Thailand is limited. Therefore, the aim of this retrospective study was to determine the demographic and clinical profile of OLP in a group of Thai patients in Oral Medicine Clinic, Naresuan University during the past 17 years.

## Material and Methods

The present study was approved by Naresuan University Institutional Review Board (NU-IRB-COA No. 530/2018). The patient archive of Oral Medicine Clinic, Dental Hospital, Naresuan University during the 2002-2018 period was retrospectively reviewed for dental record of patients with OLP. The diagnosis of patients with OLP was clinically and/or histopathologically confirmed. The diagnostic criteria proposed by van der Meij *et al.* (9) in 2003 based on the clinical and histopathologic definition of OLP by the WHO were used to identify the OLP cases. A total of 102 dental records were reviewed. Patient’s information regarding gender, age, chief symptoms, lesion distribution, clinical forms, extraoral involvement, medication use, systemic diseases and habits regarding tobacco and/or alcohol consumption, and betel nut chewing were evaluated. The data were subjected to basic variation statistical values were performed using SPSS (version 17.0; SPSS Inc., Chicago, IL, USA).

## Results

A total of 102 dental record of patients with confirmed diagnosis of OLP were retrospectively analyzed. The biopsy was performed in 48 patients (47.1%) and the left 54 patients denied.  Table 1 shows the age and gender distribution of all OLP patients. All of patients were Thai, 75 women (73.5%) and 27 men (26.5%), giving a female to male ratio of 2.8:1. Their age ranged between 20 to 81 years old with the average age of 56.4 ± 13.2 years old (average age for women is 55.9 ± 12.5, for men is 57.8 ± 15.1). The age group of 50-59 years old (27/102, 26.5%) and 60-69 years old (8/102, 7.8%) had the highest prevalence of OLP in women and men, respectively.

**Table 1:**
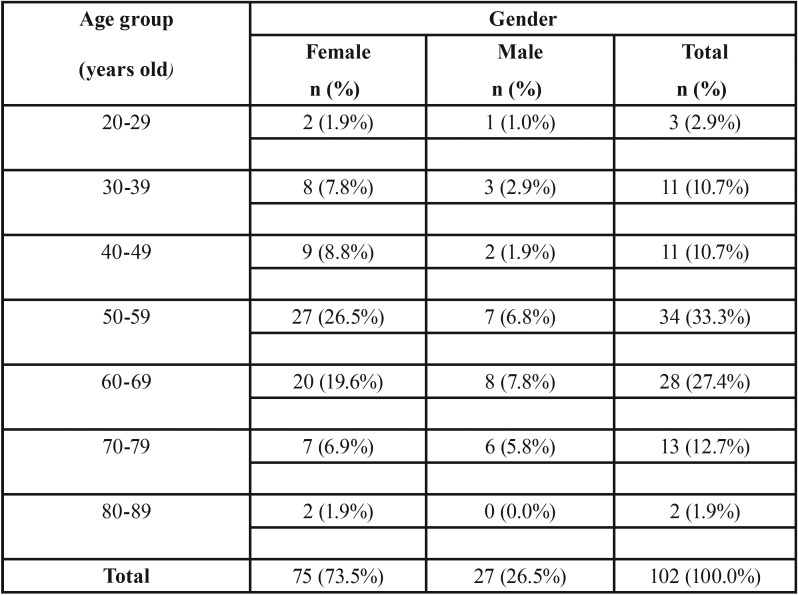
Age and gender distribution of 102 patients with OLP.

According to the medical history, 78 patients (76.5%) had the history of medication use and systemic disease. Among those, the most common systemic diseases in as descending order (number, %) were hypertension (33, 42.3%), dyslipidemia (17, 21.8%), diabetes mellitus (8, 10.3%), thyroid gland disorders (6, 7.7%), liver disease (5, 6.4%), lung disease (3, 3.8%), heart disease (3, 3.8%), epilepsy (2, 2.6%) and kidney disease (1, 1.3%). Most patients were non-smokers (100, 98%), non-drinkers (88, 86.3%) and non-betel nut chewers (100, 98%).

**Figure 1:**
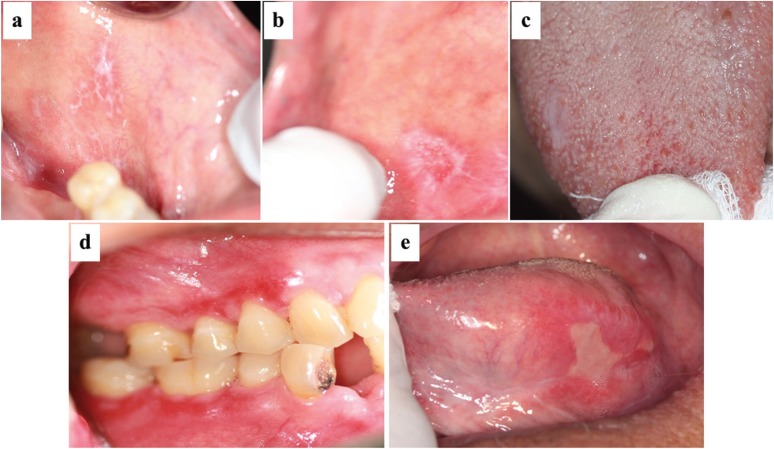
Clinical forms of oral lichen planus. (a) reticular form, (b) papular form, (c) plaque-like form, (d) atrophic form and (e) erosive/ulcerative form.

Ninety-four patients (92.2%) were symptomatic and 8 patients (7.8%) had no symptom. The OLP lesions frequently involved multiple locations (67.6%) where the buccal mucosa (79.4%) predominantly affected, followed by gingiva (64.7%), labial mucosa (25.5%), tongue (23.5%), palate (6.9%) and floor of mouth (2%). Only 2% reported with extraoral involvement which was observed on skin. Atrophic form (93.1%) of OLP were primarily found among OLP lesions, followed by reticular form (87.3%), erosive/ulcerative form (44.1%), papular form (5.8%) and plaque like form (5.8%) (Fig. 1). Multiple forms of OLP (91.2%) were frequently seen in each patient.  Table 2 shows gender distribution based on the clinical form of OLP.

**Table 2:**
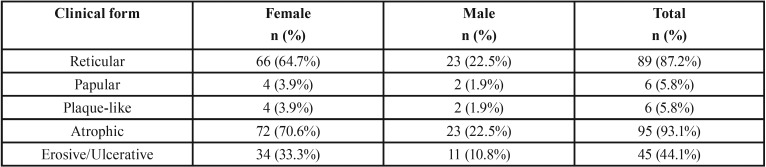
Gender distribution of OLP patients according to clinical form.

All patients had no family history of OLP or malignant transformation. Ninety-one of OLP patients (89.2%) were treated with topical steroid and only 4 patients (3.9%) were treated with a combination of topical and systemic steroid.

## Discussion

In general, the clinical profiles of Thai OLP patients in the present study are consistent with the other studies (10-14). Results of this study showed that most of patient were female (73.5%) who had 2.8 times higher prevalence of OLP than male counterpart (2.8:1 ratio). However, Munde *et al.* reported of male predominance in Indian OLP patients (female: male = 1:1.61) (6). Regarding the age of OLP patients in this study, OLP is more prevailing in the 6th decade for male and the 5th decade for female. The average age of OLP patients is 56.4 years old, which is in the same period as reported in Spain (10), Brazil (15), and China (16). However, the prevalence of OLP was peaked in the 3rd to the 4th decade of life in several studies which were lower than that found in this study (6,11,12). These dissimilarities may possibly due to the demographic difference such as the race, ethnicity, oral habits, and geography.

There was the relevant connection between OLP and systemic diseases (2,6,11,12). In the present study, as many as three quarter of patients (76.5%) had history for systemic diseases including hypertension, dyslipidemia, diabetes mellitus, thyroid gland disorder and liver disease. Their pharmacological treatment and the geriatric age may contribute to the co-morbidities and pathogenesis of OLP (14).

Although smoking, alcohol drinking, and betel nut chewing may increase the risk of OLP and malignancy (6,16), non-smokers and non-drinkers may also demonstrate malignant transformation of OLP lesions as reported by Eisen (17). In this study most patients were non-smokers (98%), non-drinkers (86.3%) and non-betel nut chewers (98%). No evidence of malignant transformation of OLP lesion was observed in these patients. A previous report in Thailand found that the malignant transformation of OLP lesion was as low as 0.2%-1.7% in Thai (18). However, since most patients normally have long-standing OLP, and perhaps a risk of malignant transformation increases. Therefore, it is mandatory of such individuals to be carefully long-term monitored by an experienced clinician (5,7).

The clinical characteristics of Thai patients demonstrated many similarities with the previous reports. For example, most patients had clinical symptoms (92.2%) such as oral discomfort, burning sensation, pain and difficulty in eating, similar to the studies in Indian (6), Brazilian (15), Chinese population (16). Multiple sites of the lesions were common in each individual (67.7%), consistent with the others (6,15,16). Buccal mucosa was the most common site of the lesion, followed by gingiva, labial mucosa, and tongue, which agreed with the previous studies (1,6,11-17). Lesions on the palate and floor of mouth were uncommon. Skin lesion was observed only in 2% of the patients in this study, whereas, Tovaru *et al.* (19) reported 25% of OLP patients had skin involvement.

The dissimilarity between this study and the other reports is the most frequent form of OLP. The atrophic form was the most common, followed by reticular form, erosive/ulcerative form, papular form, and plaque-like form. Studies in Indian (6), Chinese (16), and Romanian (19) reported the reticular form as the most frequently found, followed by erosive, and atrophic form. Eisen (17) found that the erosive form was the predominant type. Moreover, Xue *et al.* (16) reported that the erosive form showed significantly greater prevalence in old patients than the reticular or atrophic one. In the present study, most patients (91.2%) had combined clinical forms of OLP. Familial background may play important role in OLP as many cases had been reported (20). However, the incidence of family history in OLP patients was not found in the present study.

Treatments of OLP include topical and/or systemic steroids therapy, photochemotherapy, and photodynamic therapy (5). The potent topical steroids are recommended as the first drug of choice in the treatment of symptomatic OLP. Various forms of topical steroids such as orabase, ointment, solution, spray or mouthwash have been used (7). Asymptomatic OLP need not to be treated, but follow-up is recommended. Patients in this study were treated by topical (89.2%) and a combination with systemic steroids (3.9%). In addition, removal of irritation (i.e. sharp cusps, broken restorations and non-opposing tooth) and control oral hygiene is recommended and found to be beneficial in the management of OLP. Long-term follow-up should be reserved for patients with complicated OLP and those who are unresponsive to treatment. Tissue areas that do not respond to treatment may need further evaluation and possibly future biopsy. Biopsy is indicated if malignancy change is suspected (7).

In conclusion, the present study was performed to illustrate the characteristics of oral lichen planus in a group of Thai patients in the north Thailand. The results of the study indicated that most of characteristics are in accordance with previous studies. Since, OLP is a chronic inflammatory oral mucosal disease with high recurrence rate, long-term follow-up is necessary to evaluate the exacerbation and malignant transformation.
